# Supplementary material for: Molecularly Imprinted Polymer-Amyloid Fibril-Based Electrochemical Biosensor for Ultrasensitive Detection of Tryptophan
Source: Biosensors (Basel). 2022 May 2;12(5):291. doi: 10.3390/bios12050291 (PMC9139163; doi:10.3390/bios12050291)
Supplement: Supplementary file 1 [file biosensors-12-00291-s001.zip › biosensors-1688938-supplementary.pdf]

biosensors

Supporting Materials

Molecularly Imprinted Polymer-Amyloid Fibril-Based Electrochemical Biosensor for Ultrasensitive Detection of Tryptophan

Ibrar Alam [1], Benchaporn Lertanantawong [2], Thana Sutthibutpong [3,4,5], Primana Punnakitikashem [6,7] and Piyapong Asanithi [1,3,4,5,*]

1 Nanoscience and Nanotechnology, Faculty of Science, King Mongkut's University of Technology Thonburi, Bangkok 10140, Thailand; ibrar.alam@mail.kmutt.ac.th
2 Department of Biomedical Engineering, Faculty of Engineering, Mahidol University, Salaya, Nakorn, Pathom 73170, Thailand; benchaporn.ler@mahidol.ac.th
3 Department of Physics, Faculty of Science, King Mongkut's University of Technology Thonburi, Bangkok 10140, Thailand; thana.sut@mail.kmutt.ac.th
4 ThEP Center, Commission of Higher Education, 328 Si Ayuthaya Rdad, Bangkok 10400, Thailand
5 Theoretical and Computational Science Center (TaCS), Science Laboratory Building, Faculty of Science, King Mongkut's University of Technology Thonburi, Bangkok 10140, Thailand
6 Siriraj Center of Research Excellence in Theranostic Nanomedicine, Faculty of Medicine, Siriraj Hospital, Mahidol University, Bangkok 10700, Thailand; primana.pun@mahidol.ac.th
7 Department of Biochemistry, Faculty of Medicine, Siriraj Hospital, Mahidol University, Bangkok 10700, Thailand
* Correspondence: piyapong.asa@kmutt.ac.th

Material characterization:

Preparation of PANI-no AF and PANI-AF

PANI was obtained by oxidizing 100 mM aniline with an aqueous solution of 10 mM iron (III)chloride in 10 mM HCl at room temperature. After 5 min of stirring, the solution was kept at room temperature overnight. PANI was recovered by filtration and washed several times with DI water. PANI-AF was produced in the same way as PANI-no AF but in the presence of 2 mM AF.

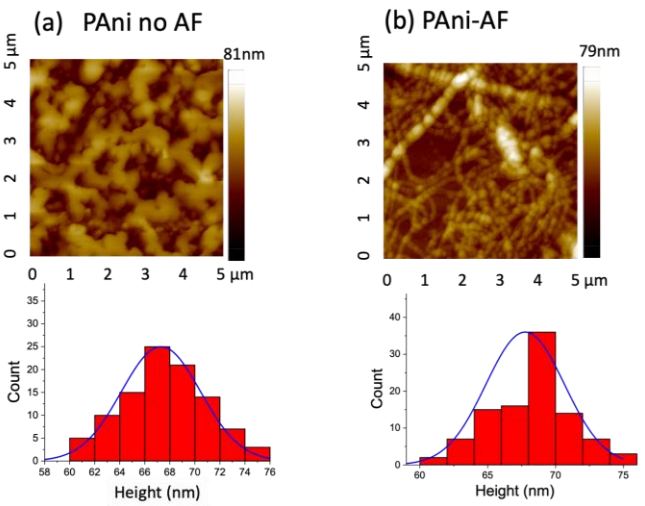

Figure S1. AFM for investigating the effect of AF on aniline polymerization. (**a**) Aniline polymerization without AF (PANI no AF) provides a cluster-like structure. (**b**) Aniline polymerization in the presence of AF (PANI-AF) clearly shows PAni -covered surface of AF.

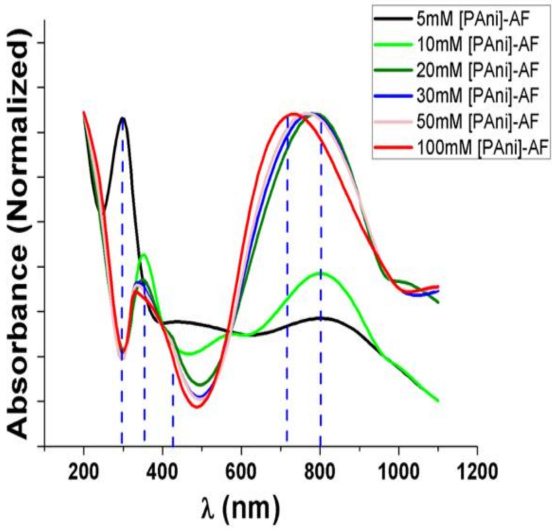

Figure S2. Determining the concentrations of aniline for polymerization around AF.

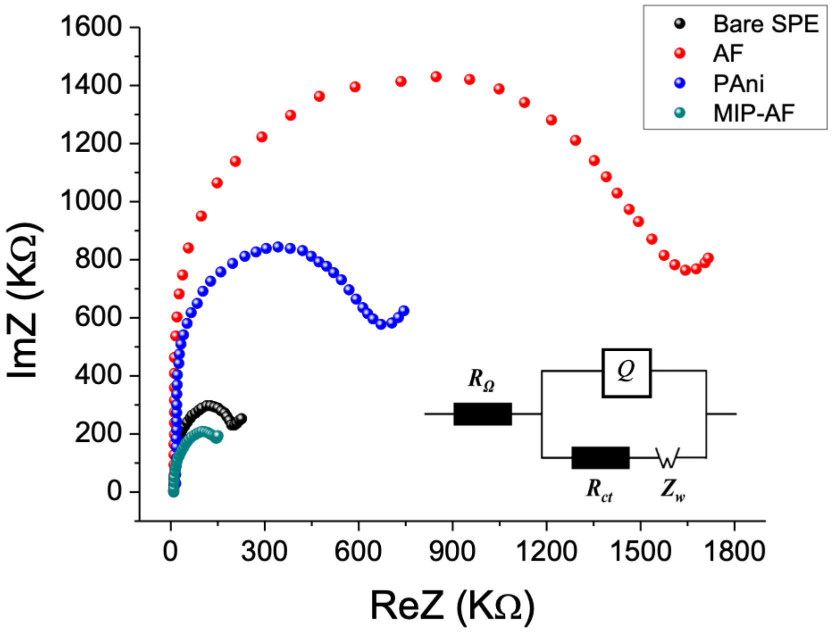

Figure S3. EIS Nyquist plot for characterizing MIP-AF development.
